# Treatment of Hemorrhoids by Hypodermic Injection of Carbolic Acid

**Published:** 1879-09

**Authors:** 


### TREATMENT OF HEMORRHOIDS BY HYPODERMIC INJECTION OF CARBOLIC ACID.

Dr. Andrews' conclusions from 3,300 cases treated in this way, are:

1. Inject only internal piles.
2. Use diluted forms of the remedy at first, and stronger ones only when these fail.
3. Treat one pile at a time, and allow from four to ten days between the operations.
4. Inject from one to six drops, having smeared the membranes with cosmoline to guard against dripping. Inject very slowly, and keep the needle in place a few moments to allow the fluid to become fixed in the tissues.
5. Confine the patient to bed the first day, and also subsequently, if any severe symptoms appear. Prohibit any but very moderate exercise during the treatment.

With these precautions, he thinks this operation will be of permanent value. The operation is performed in the following way: The pile is exposed to view, and the anus smeared with an ointment, to prevent smarting in case the fluid should chance to drop. The operator then takes a sharp-pointed hypodermic syringe, charged with the carbolized liquid (which has been used in varying strength, from one part of the crystallized acid to thirty of olive oil, or glycerine up to equal parts,) and slowly throws a few drops into one of the piles. The needle is left in the puncture a few moments, to prevent the fluid from running out, and to allow it to become fixed in the tissue. The pile turns white, and in the most successful cases withers away without pain, suppuration, or sloughing.

Only one pile is treated at a time, and about a week is allowed between the sessions. Most of the cases thus operated upon, suffer a sharp, temporary smarting, and a few have a terrible and prolonged agony. The majority are cured, however, without interrupting the patient's business.—*Chicago Medical Journal.*

